# Imaging of acute musculoskeletal infections in children and their differential diagnoses

**DOI:** 10.1007/s00247-026-06564-8

**Published:** 2026-03-16

**Authors:** Morgan N. McLuckey, Justine M. Kemp, Arthur B. Meyers

**Affiliations:** 1 Department of Medical Imaging, Ann & Robert H. Lurie Children’s Hospital of Chicago, Chicago, United States; 2 Department of Radiology, Northwestern University Feinberg School of Medicine, Chicago, United States; 3 Cincinnati Childrens Hospital Medical Center, 3333 Burnet Ave, ML 5031, Cincinnati, OH 45229 United States; 4 University of Cincinnati Medical Center, Cincinnati, United States

**Keywords:** Pediatric, Musculoskeletal, Emergency, Imaging, Infection, Osteomyelitis

## Abstract

Musculoskeletal complaints are a frequent cause of emergency department visits in pediatric patients. While many of these visits are due to acute trauma, infection is also common and can cause substantial morbidity. This article provides a review of acute pediatric musculoskeletal infections with an additional emphasis on important differential diagnoses to consider. The scope includes cellulitis, abscess, necrotizing soft tissue infections, pyomyositis, infectious tenosynovitis, septic bursitis, septic arthritis, and osteomyelitis. Important differential considerations for these musculoskeletal infections will also be briefly reviewed.

## Introduction

Musculoskeletal infections are a frequent cause of pediatric emergency department visits and may pose a diagnostic challenge. This is particularly true in young children who often present with non-specific symptoms such as fever, limp, or decreased use of an extremity. There is a wide range of possible etiologies for these presentations, some reflecting underlying conditions which are benign and self-limiting (e.g., transient synovitis) while others require urgent treatment (such as osteomyelitis and septic arthritis) to avoid long-term complications. In this review, we will focus on musculoskeletal infections in children and important differential diagnostic considerations (Table 1).

**Table 1:** Infectious musculoskeletal emergencies in children and differential diagnostic considerations

**Musculoskeletal infections** Cellulitis Abscess Necrotizing soft tissue infections Pyomyositis Infectious tenosynovitis Septic bursitis Septic arthritis Osteomyelitis
**Important differential diagnosis considerations**
Transient synovitis Rheumatologic conditions - Juvenile idiopathic arthritis (JIA) - Chronic non-bacterial osteomyelitis (CNO)
Malignancy - Leukemia - Metastases (e.g., neuroblastoma) - Primary bone tumors (e.g., osteosarcoma, Ewing sarcoma) Metabolic/nutritional disorders Scurvy

## Cellulitis

Skin and soft tissue infections (SSTIs) are exceedingly common in the acute setting with a rising incidence over the last decade. The majority of pediatric SSTIs are encountered in the emergency department [1] and encompass a wide spectrum of pathologic conditions. Chief among these is cellulitis, a non-necrotizing infection of superficial soft tissues [2, 3]. The superficial soft tissues include the skin, subcutaneous fat, and superficial (or membranous) fascia [4]. This fascia, which is contained within the subcutaneous fat, should be differentiated from the deep fascia (which includes the investing layer around the muscle and the intramuscular fascia) [5]. There are an estimated 14.5 million cases in the USA per year [6]. It is challenging to enumerate the incidence of causative pathogens given that cellulitis is typically not culturable; however, *Staphylococcus aureus* and beta-hemolytic Streptococci appear to be the most common [2]. Risk factors include essentially anything that disrupts the skin allowing for inoculation, such as trauma, wounds, burns, skin lesions, surgery, or insect bites [2].

Diagnosis is primarily clinical and the majority of SSTIs do not warrant imaging [7]. Identification of complicated cellulitis, including presence of abscess or underlying foreign body, or investigation for alternate causes of soft tissue swelling (such as fracture or deep venous thrombosis (DVT)) may prompt radiologic evaluation [2]. Ultrasound (US) is well-suited and typically sufficient to exclude the presence of a superficial abscess [8], though magnetic resonance imaging (MRI) may have a role in deeper infections [9]. Radiography or US may have utility depending on the suspected foreign body (wood, plastic, or metal) [10]. Computed tomography (CT) is typically not indicated [9]. Advanced imaging to investigate for concomitant osteoarticular infection is usually unnecessary in the upper extremities as the incidence of this is low, though it may be appropriate in the lower extremities [11].

**Fig. 1:**
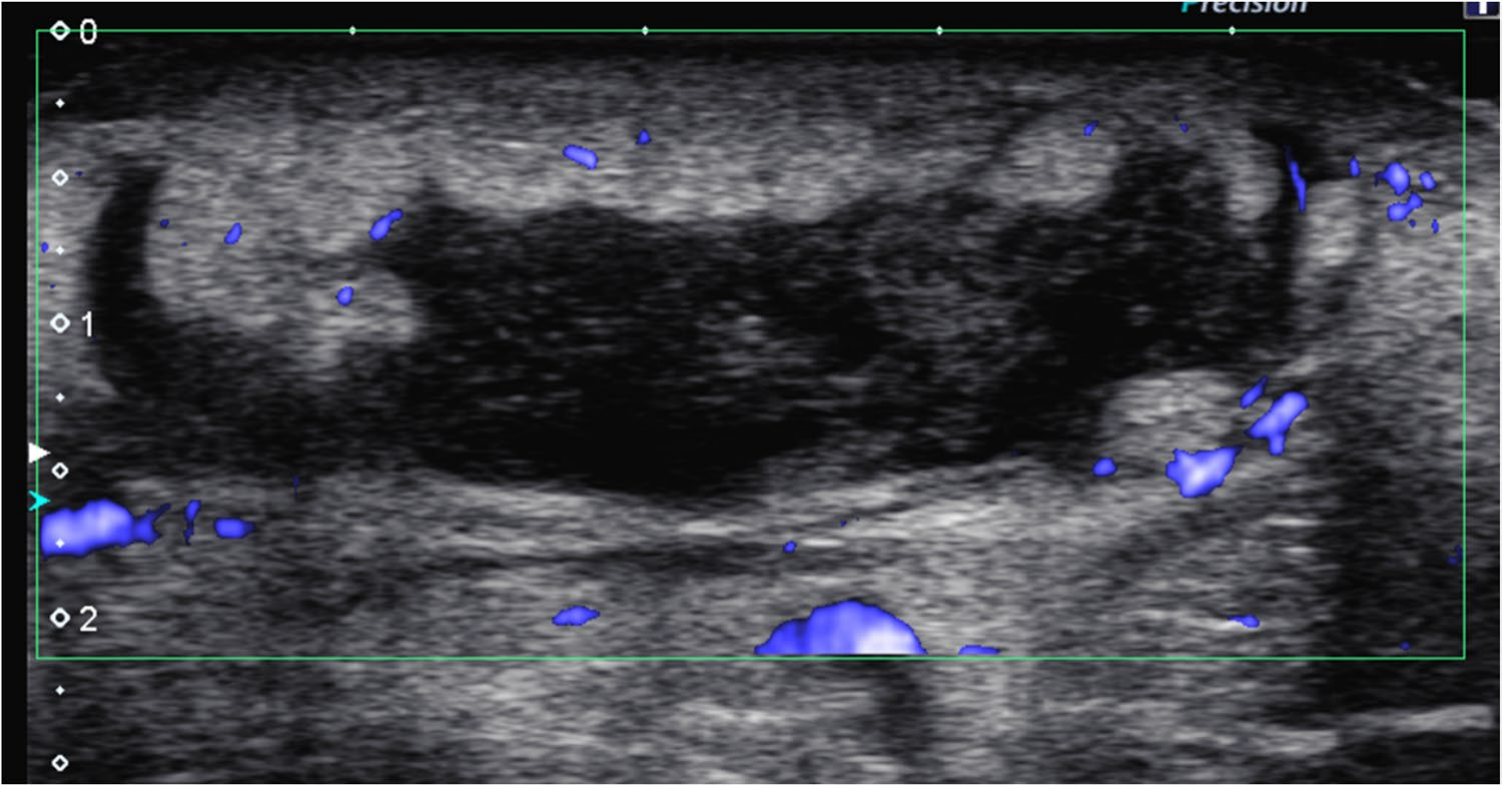
Cellulitis and abscess on ultrasound. A 14-year-old girl who presented to the emergency department with proximal right forearm swelling in the area of bug bites. Power Doppler transverse image of the volar soft tissues of the area shows a large 5-cm hypoechoic collection with peripheral hyperemia. Note the surrounding soft tissues show increased echogenicity

On radiography, uncomplicated cellulitis will demonstrate non-specific soft tissue swelling with effacement of fat planes [9]. US will show skin thickening, increased echogenicity in the subcutaneous fat, subcutaneous edema with “cobblestoning,” and hyperemia (Fig. 1) [9]. MRI is not needed to diagnose superficial infections; however, it is warranted when there is concern that an infection may involve deeper tissues as it can show the extent of both superficial and deep infections. In cellulitis, MRI demonstrates thickening of the superficial soft tissues with reticular and/or confluent hyperintense signal on fluid-sensitive sequences with corresponding hypointense signal on T1-weighted sequences [3, 9]. If contrast is given, these areas will variably enhance thus differentiating cellulitis from bland edema [5, 9]. Cellulitis will also show some degree of restricted diffusion on diffusion-weighted imaging (DWI), as opposed to bland edema which will show facilitated diffusion [12]. Though poorer in contrast resolution when compared to MRI, CT will similarly show areas of confluent and/or reticular subcutaneous attenuation which variably enhances [9]. The deeper soft tissues (deep fascia, muscle) are spared in cellulitis alone.

Antibiotics, usually on an outpatient basis, are the treatment of choice for non-purulent SSTIs [7], though some complicated forms of cellulitis (abscess, foreign body, lymphadenitis) may require additional or more intensive therapy and/or procedures [2, 5]. Abscesses are discussed in further detail in the next section. Lymphadenitis is infectious inflammation and enlargement of the lymph nodes and is readily identified on US by the presence of enlarged and morphologically abnormal lymph nodes with hyperemia. While treatment with oral antibiotics is usually sufficient [13], larger lymph node size and younger patient age have been associated with use of intravenous antibiotics, return visits, and need for surgical drainage [14]. A unique infection, cat scratch disease (caused by *Bartonella henselae*), can be suggested by characteristic lymphadenitis of the medial epitrochlear node of the elbow [15], though it can also present more systemically with osteomyelitis and granulomas [16].

## Abscess

An abscess is a walled-off purulent collection surrounded by a capsule of macrophages, fibrin, and granulation tissue [4]. Phlegmon is an inflammatory soft tissue mass, an intermediate state on the spectrum between cellulitis and abscess [5]. The Society of Skeletal Radiology (SSR), however, has recommended against using the term “phlegmon” in radiology reports to describe coalescent areas of infection as its use does not lead to a change in clinical management and may cause confusion, and to instead use “cellulitis” or “myositis” without abscess [4]. Abscesses are most commonly caused by *S. aureus*, followed by *Streptococcus* species and coagulase-negative *Staphylococcus*. Methicillin-resistant *S. aureus* (MRSA) with production of the Panton-Valentine leukocidin (PVL) toxin, which has added virulence due to leukocyte destruction and tissue necrosis, may cause recurrent SSTIs and abscesses [2, 17]. While the diagnosis of abscess can be made based on history and physical examination, significant overlap with the clinical appearance of uncomplicated cellulitis presents a challenge [18]. Imaging better identifies abscess in the setting of SSTI, determines size and location(s) of the collection, and helps plan the necessary drainage [2].

Radiographs may show only the soft tissue swelling also seen in cellulitis, though mass-like attenuation can be present in the case of large abscesses. Air-fluid levels are uncommon [5]. US, often performed at the point-of-care, is highly accurate in the diagnosis of abscess [18]. Findings include a heterogeneous or hypoechoic fluid collection, with or without internal septations, which may have hyperemic and hyperechoic rims surrounded by cellulitic or phlegmonous tissue (Fig. 1). Posterior acoustic enhancement can also be seen. Dynamic compression may be helpful in demonstrating swirling of internal contents and more confidently differentiating abscess from phlegmon. Gas bubbles will manifest as echogenic foci with dirty shadowing [5, 9].

While US is usually sufficient for superficial abscesses, MRI has the highest sensitivity and specificity for detection of abscess and will allow the detection of deep soft tissue and even intra-osseous abscesses. It will be seen as a collection with heterogeneous, hyperintense signal on fluid-sensitive sequences with corresponding hypo- to isointense signal on T1-weighted sequences [5, 9]. These central contents will restrict diffusion due to high viscosity of the purulent material [12] and the surrounding rim avidly enhances with contrast; these features are key in differentiating an abscess from cellulitis or phlegmon. The rim of granulation tissue may also show intrinsic T1 shortening (penumbra sign) in subacute or chronic infections [5]. Some studies have explored abscess detection without the use of intravenous contrast, relying on DWI [19, 20]. Comparable diagnostic accuracy has been demonstrated, suggesting that DWI is vital when contrast cannot be given, though it should also be noted that DWI is prone to artifacts and is particularly susceptible to motion (an issue in non-sedated pediatric patients). Corresponding to the features described on MRI, on CT, abscess will appear as a hypoattenuating collection with an enhancing rim, usually in the context of other soft tissue infections [5]. US and MRI are preferred to CT given their lack of ionizing radiation [3]. If CT is performed, contrast is necessary.

Treatment consists of incision and drainage or percutaneous drainage and antibiotics [2, 21], noting that some recurrent abscesses or abscesses in specific locations may reflect an infected cyst (e.g., branchial cleft, epidermal inclusion, pilonidal) instead requiring enucleation. Analysis of the drained fluid can also tailor the antibiotic therapy. Some caution may be warranted in describing abscesses as “drainable” in radiology reports, as whether a collection can or should be drained is dependent on the clinical context and it can be difficult to determine whether infected material is drainable based on imaging features [4].

## Necrotizing soft tissue infections

Necrotizing soft tissue infections (NSTIs) are an important subset of soft tissue infections owing to their high morbidity and mortality and need for urgent diagnosis and intervention. NSTIs, whether superficial or deep, are characterized by rapid progression of microvascular occlusion, ischemia, and liquefactive necrosis [22, 23]. They are accompanied by significant morbidity and mortality [24]. NSTIs have been grouped into several types based on causative microorganism and location. Type I infections are polymicrobial, comprise most NSTIs, and tend to involve the trunk. Type II infections are typically monomicrobial (group A *Streptococcus*, sometimes in association with *Staphylococcus aureus*) and more commonly involve the extremities. Type III infections are monomicrobial and gram-negative, such as *Clostridium* or *Vibrio* species. Type IV infections are fungal [5, 22].

In contrast with adults in whom type I infections are most common, type II infections predominate in children [25]. Data on incidence in children is sparse, though a large prospective population surveillance study estimated 0.08 per 100,000 children per year for type II infections [26]. Documented etiologies in neonates include omphalitis, iatrogenic (minor trauma from monitoring leads, intravenous line infections, intramuscular injections), surgical complications, varicella, and immunodeficiency [27, 28]. In children, leading causes and predisposing factors include trauma, malnutrition, and varicella [28–30].

NSTI is principally a clinical diagnosis, and radiologic findings are often non-specific though imaging may be requested for surgical planning or challenging cases. The Laboratory Risk Indicator for Necrotizing Fasciitis (LRINEC) score is a clinical decision tool that uses laboratory data (C-reactive protein, total white cell count, hemoglobin, sodium, creatinine, glucose) to stratify risk of a NSTI on a 13-point scale [31]. A score of 6–7 is considered moderate risk and a score of 8 or more is high risk. This score has been integrated with MRI [32] to further improve positive and negative predictive values (82% and 79%, respectively) in differentiating necrotizing from non-necrotizing fasciitis.

**Fig. 2:**
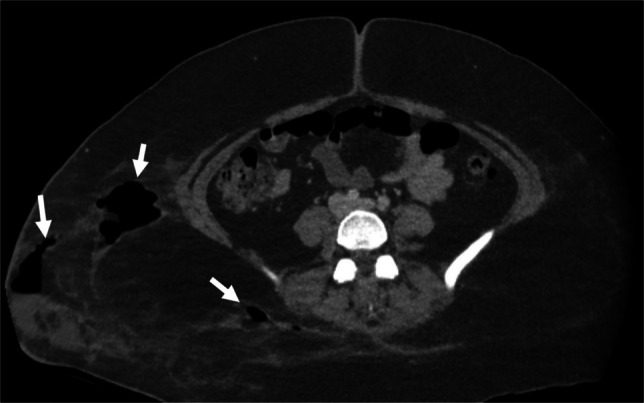
Necrotizing fasciitis. A 15-year-old girl with a history of a motor vehicle collision and abrasion to the right flank 1 month prior presented to the emergency department with concerns for a wound infection. Axial CT with contrast shows multiple air-filled collections within the soft tissues of the right flank (*white arrows*). The most lateral collection has an air-fluid level

In the absence of traumatic or iatrogenic causes, gas extending along fascial planes is diagnostic [33]. However, not all NSTIs are gas-forming, and the absence of gas does not exclude NSTI [22]. CT is preferred over MRI given its high sensitivity for gas, ability to assess extent of disease, ease of access, and rapid acquisition time (Fig. 2). Focal subcutaneous edema and fascial thickening are the most common though non-specific CT findings. MRI is superior in its depiction of dermal, soft tissue, and fascial thickening, though these findings are also present to some degree in non-necrotizing fasciitis [33]. Research has attempted to define discriminating features of NSTIs on MRI. Some criteria that have been used to distinguish necrotizing from non-necrotizing infectious fasciitis include deep fascial thickening (≥3 mm), non-enhancing areas in the deep fascia, extensive involvement of the deep fascia, and involvement of ≥3 compartments in one extremity [34, 35]. The absence of signal alteration in the deep fascia appears to have a high negative predictive value [34, 35]. However, it is important that treatment is not delayed for the performance of imaging.

## Pyomyositis

Pyomyositis is a bacterial infection of the muscle with suppuration/liquefaction. It is more common in tropical climates where it has been estimated to account for almost one in 25 surgical hospital admissions, though it can also occur in more temperate climates [36]. It is more common in the pediatric than the adult population [5]. There is evidence to suggest that mechanical trauma at the time of bacteremia or the presence of underlying health conditions (HIV, diabetes mellitus) may predispose to pyomyositis [9, 36]. The most frequently involved sites are the iliopsoas, thigh, and lower leg, and infection is usually caused by *S. aureus* or *S. pyogenes* [3, 5, 36]. Bacterial pyomyositis has been described in three clinical stages. Stage 1 usually passes before clinical presentation and consists of malaise, low-grade fever, dull or cramping pain, and lack of purulent material. Stage 2 is characterized by frank pus in the muscle with severe pain and swelling and is typically when patients present. Stage 3 evolves to high fever with sepsis and systemic complications [36, 37].

**Fig. 3:**
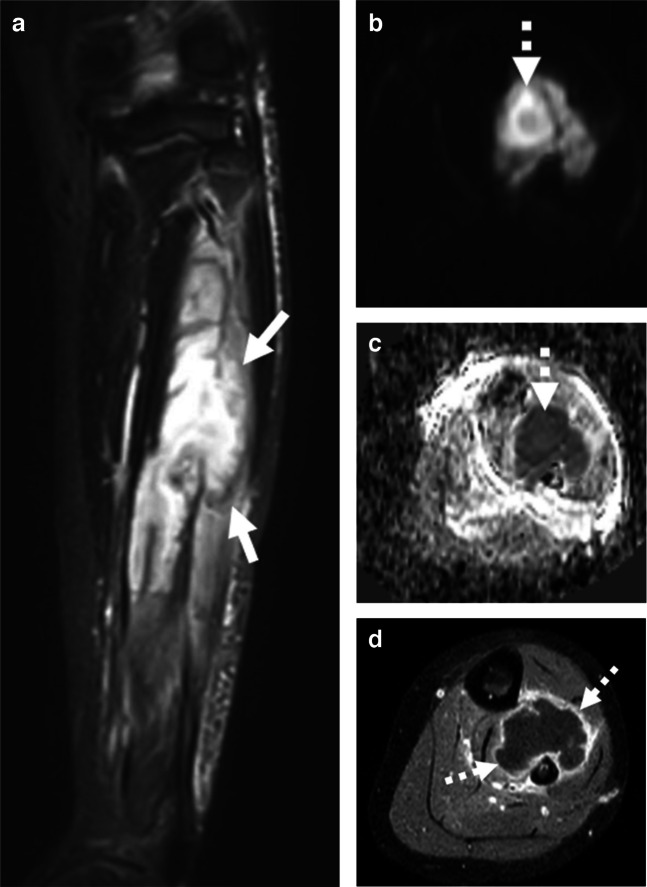
Pyomyositis with suppuration. A 10-year-old boy with soft tissue laceration from another player’s cleat during a football game presenting with lower extremity pain. **a** Coronal STIR image shows a globular, T2 hyperintense collection in the musculature of the lower leg (solid arrows). Axial diffusion-weighted image (DWI) (**b**) and corresponding apparent diffusion coefficient (ADC) image (**c**) demonstrate increased focal increased signal on the DWI image with corresponding low signal on the ADC image, consistent with an abscess (*dashed arrows*). **d** Axial T1 FS post-contrast image shows a thick, enhancing rim around the collection (*dashed arrows*), which is centered in the deep lower leg musculature

MRI with contrast is the imaging modality best suited to characterize pyomyositis [7]. MRI will show enlargement of the muscle with variable signal, depending on the clinical stage. In the early (stage 1) infection, there will be heterogeneous T2 hyperintense signal with minimal T1 hyperintense signal; in the later purulent phases, this will become T2 hyperintense and T1 hypointense [9, 37]. Also at this stage, single or multiple rim-enhancing purulent collections will have developed. They will restrict diffusion [12], having the same appearance as abscess in the superficial soft tissues (Fig. 3) [9]. This appearance can be contrasted with that of viral myositis on MRI, which will show more patchy and streaky T2/short tau inversion recovery (STIR) hyperintense and T1 hypo- to isointense signal throughout the affected muscle [37, 38].

CT with contrast can be used alternatively, but its inferior soft tissue contrast may underestimate disease in the early stage [9]. CT will show muscle enlargement with effacement of fat planes, heterogeneous attenuation, and rim-enhancing fluid collection when suppuration is present [5]. US may demonstrate the liquefied contents as a hypoechoic or complex intramuscular collection without internal vascularity and surrounding hyperemic tissues, with a differential diagnosis of hematoma [5, 9]. However, deeper infection (such as in the pelvis or the thigh in larger patients) may limit use of US. Prompt drainage of purulent material is recommended along with intravenous antibiotics. Repeat imaging may be useful to assess for persistent liquified collections in the setting of continued fever despite treatment [7].

## Infectious tenosynovitis

Infectious tenosynovitis is an infection of the tendons and surrounding synovial sheath [12]. The largely closed nature of the paratendinous synovial compartment predisposes to infection [39]. The most commonly involved sites are the hands/wrists and feet/ankles due to high frequencies of direct inoculation by trauma. Other mechanisms include direct spread from adjacent infection or hematogenous dissemination [40]. The array of potential microbiology is vast and depends on the exposure. As with many of the earlier infections discussed, *S. aureus* and *Streptococci *species are common [40]. Bite wounds may introduce unique pathogens, such as mixtures of aerobic and anaerobic bacteria and *Eikenella corrodens* (especially with clenched-fist injuries) in human bites and *Pasteurella multocida* in animal bites [39, 41]. Children sustain animal bites more commonly than adults [41].

Symptoms include pain, swelling, and erythema of the affected area. Infection with *P. multocida* will present more intensely and acutely (within 12–18 h) [41]. Mycobacterial infections may have a more indolent course [39, 40]. Tenosynovitis of the flexor tendons of the hand may result in slight flexion of the affected digits [40]. Permanent contracture of the affected tendon and muscle may result from delayed or inadequate treatment; and therefore, a high index of suspicion should be maintained by the clinician and the radiologist [39].

**Fig. 4:**
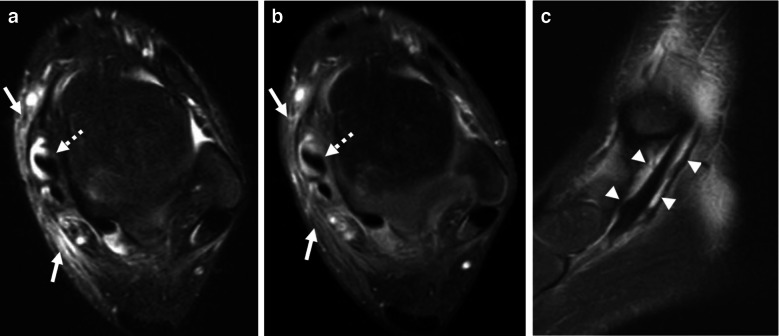
Cellulitis and infectious tenosynovitis. A 10-year-old boy with a skin infection of the medial ankle presenting with increased swelling and fever. **a** Axial T2W fat-suppressed (FS) and (**b**) axial T1W FS post-contrast images of the right ankle show increased signal on the T2W FS image which enhances on the post-contrast sequence (*solid arrows*) consistent with cellulitis. Also note the increased signal and enhancement within the posterior tibialis tendon sheath (*dashed arrow*). **c** Sagittal T2W FS image shows distention of the posterior tibialis tendon sheath with hyperintense material (*arrowheads*), consistent with fluid and synovial thickening signifying tenosynovitis. Cultures from incision and drainage revealed methicillin-resistant *Staphylococcus aureus* (MRSA)

US and MRI are the best imaging modalities to identify and characterize infectious tenosynovitis. Findings will include fluid distention of the tendon sheaths (usually intermediate T1 signal and hyperintense fluid-sensitive signal [42]) with synovial thickening (Fig. 4). US will additionally demonstrate hyperemia and MRI will demonstrate avid post-contrast enhancement of the synovium [12, 39]. The underlying tendons may appear normal or mildly thickened [42]. DWI may also show restricted diffusion within the tendon sheath if the contents are highly viscous [12]. Additional findings of periosteal reaction and osseous erosions may also be present and can be characterized on radiography or MRI (and, to a lesser extent, US) [39]. Inflammatory or rheumatologic tenosynovitis is the primary differential consideration, and imaging features should be interpreted in the context of the clinical history. Ultimately, fluid sampling may be necessary as the sterility of fluid cannot be determined by imaging alone [39].

The unique anatomy of the hand necessitates special consideration in the context of infectious tenosynovitis. The flexor tendon sheaths of the thumb (flexor pollicis longus) and little finger (flexor digitorum of the fifth digit) almost always communicate with the radial bursa and ulnar bursa, respectively [43]. These bursae also usually communicate with one another or may rupture into the deeper space of Parona [42, 43]. Therefore, infection may spread between the radial and ulnar hand and into deep spaces, known as a “horseshoe abscess” [42]. Awareness of this unique anatomy may prove particularly useful in the context of US imaging, when the entirety of the affected anatomy may not be imaged without specific direction.

## Septic bursitis

Septic bursitis, infection of the synovial bursae, can result from penetrating trauma, direct spread of adjacent infection, or hematogenous spread [12]. Commonly-affected areas include the prepatellar, olecranon, and calcaneal bursae [12, 40]. The usual organisms are *S. aureus* and *Streptococci*; other organisms such as mycobacteria are uncommon but possible [40]. Patients will experience pain and erythema of the affected area with associated soft tissue swelling and often cellulitis. Systemic signs of infection are more common when deeper bursae are affected. Septic bursitis should be differentiated from aseptic bursitis, which usually presents later in the course of the disease due to milder symptoms [40].

**Fig. 5:**
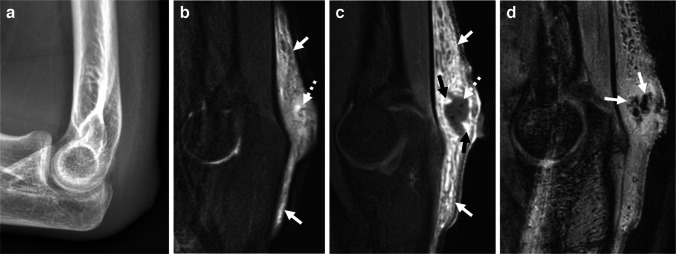
Septic bursitis. A 17-year-old male presented with cellulitis and septic olecranon bursitis after getting a tattoo. **a** Lateral radiograph of the elbow shows soft tissue swelling overlying the olecranon with reticular subcutaneous edema. Sagittal T2W FS (**b**) and sagittal T1W FS post-contrast (**c**) MR images show extensive T2 hyperintense reticular signal which enhances on the post-contrast image (*solid white arrows*), consistent with cellulitis. Additionally, there is a focal heterogenous collection at the location of the olecranon bursa that shows central non-enhancement (*dashed arrows*) and nodular peripheral hyperenhancement (*black arrows* in **c**) consistent with septic bursitis. **d** Sagittal gradient echo image shows central foci of blooming artifact (*arrows*) indicative of gas. There was no concurrent septic arthritis. Incision and drainage were performed and cultures were positive for oxacillin-resistant *Staphylococcus aureus*. Symptoms resolved after drainage and antibiotics

MRI and US are best suited to characterize septic bursitis, with MRI performing better in the setting of deeper infection. Findings will include fluid distention of the affected bursa with or without overlying cellulitis, thickening of the synovial lining, hyperemia (on US), and avid post-contrast enhancement of the synovium (on MRI) (Fig. 5) [12, 40]. Restricted diffusion will be seen in the infected bursa due to purulent contents. However, in the setting of concurrent hemorrhage, T2 shine through and susceptibility artifacts may also be present and complicate the imaging appearance [12]. Imaging can also serve to exclude or rule in a concurrent septic arthritis in the form of a joint effusion. The radiologist cannot confidently differentiate septic from aseptic bursitis, but imaging may facilitate procedural planning for sampling and drainage.

## Septic arthritis

Joint effusions, particularly of the hip and knee, are often seen in the acute setting and require differentiation between infectious, non-infectious, reactive, and rheumatic etiologies. This section will focus primarily on differentiating septic arthritis from transient synovitis.

Septic arthritis, a bacterial infection of the joint, may lead to devastating complications and should be promptly diagnosed. The incidence in children is about half that of hematogenous osteomyelitis [17]. Vaccination has led to a marked decrease in isolated septic arthritis due to *Haemophilus influenzae* and most cases are now due to *S. aureus* or *S. pyogenes*, which are often associated with osteomyelitis [44]. Transient synovitis is a benign and temporary inflammation of the hip [45]. This represents the most common cause of hip pain in children, the etiology of which is poorly understood [46]. Kocher et al. and subsequent authors have attempted to establish reliable clinical predictors differentiating septic arthritis from transient synovitis including inability to bear weight, fever, leukocytosis, and elevated erythrocyte sedimentation rate (ESR) and C-reactive protein (CRP) with CRP ≥20 mg/L as the strongest predictor of septic arthritis [47–51], though the consequences of a false negative diagnosis present a challenge to the clinician. The probability of septic arthritis increases with each modified Kocher criteria present on a scale of 0−5 (16.9%, 36.7%, 62.4%, 82.6%, 93.1%, and 97.5%) [49].

**Fig. 6:**
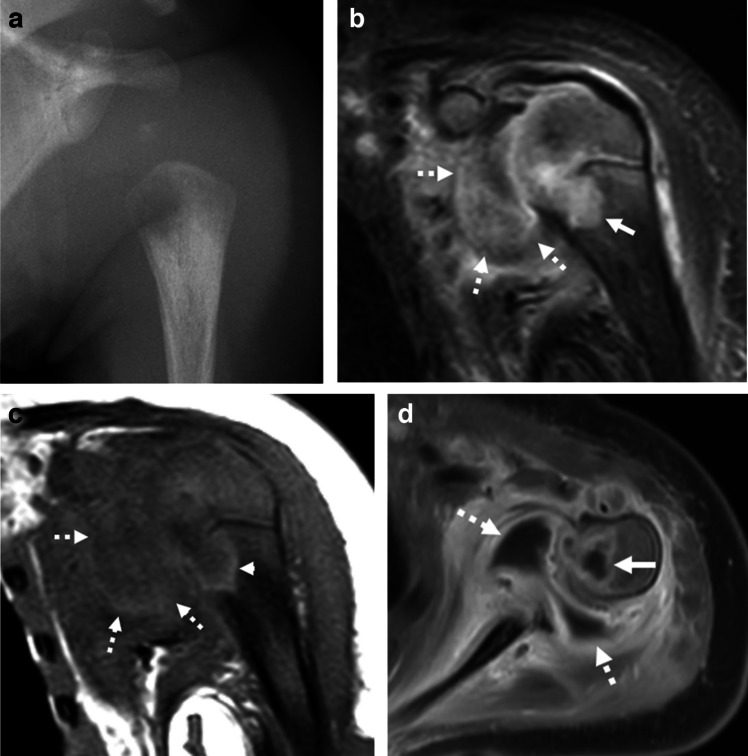
Osteomyelitis and septic arthritis. A 1-month-old boy presenting with decreased use of the left arm. **a** Frontal radiograph of the left shoulder shows a lytic lesion in the proximal left humeral metaphysis. Coronal T2W FS (**b**) and T1W (**c**) MR images show a complex collection within the proximal humeral metaphysis (*solid arrow*), which involves the physis. There is a large glenohumeral joint effusion (*dashed arrows*). Notice the peripheral increased signal on the T1 image (*arrowhead* in part **c**) consistent with the penumbra sign. **d** Axial T1W FS post-contrast image shows rim enhancement of the proximal humeral collection, consistent with intraosseous abscess (*solid arrow*), and thick peripheral enhancement of the shoulder joint (*dashed arrows*) related to the septic arthritis

Radiographs should be performed to exclude other causes of hip pain. Ultrasound is highly sensitive for the presence of effusion, though it cannot differentiate an aseptic from a septic joint [52]. Yang et al. attempted to distinguish these pathologies on MRI, finding that transient synovitis often has an associated asymptomatic effusion in the contralateral hip joint, while septic arthritis is almost always unilateral; septic arthritis exclusively demonstrated bone marrow signal intensity alterations and erosions and was more commonly associated with adjacent soft tissue abnormal signal and enhancement (Fig. 6); and both typically show synovial thickening and enhancement [46]. While MRI may provide more confidence in a diagnosis of either transient synovitis or septic arthritis, it ultimately does not exclude a septic joint and aspiration is often needed. Lyme arthritis is an additional confounder in endemic areas and is often clinically indistinguishable from septic arthritis. Proposed MRI features differentiating Lyme arthritis from septic arthritis include larger effusions with greater synovial thickening and adenopathy in Lyme arthritis, and myositis, subcutaneous edema, and osseous erosions in septic arthritis [44, 53].

There are several notable features of joint effusions which should prompt the radiologist to also consider juvenile idiopathic arthritis (JIA) in the differential diagnosis of a joint effusion. Multifocal joint involvement favors JIA over septic arthritis. Small, uniform filling defects within a joint effusion, referred to as rice bodies, are an indication of a chronic process which in children is most commonly due to JIA. Rice bodies are not a feature of acute septic arthritis but can also be seen in low-grade chronic atypical infections (e.g., mycobacterial infections) [54]; small intra-articular bodies can also be seen in the setting of primary synovial chondromatosis. It has also been suggested that low signal intensity synovial thickening on MRI may indicate JIA [55].

## Osteomyelitis

Osteomyelitis is one of the most significant non-traumatic musculoskeletal emergencies encountered in children due to its relative frequency and the consequences of treatment delay. Hematogenous osteomyelitis is the most common form of osteomyelitis in children, though infection of the bone may also occur from contiguous extension through soft tissue. Incidence has been estimated as 2–13 per 100,000 in developed countries and 43–200 per 100,000 in developing nations [56]. Osteomyelitis is acute if diagnosed within 4 weeks after onset of clinical symptoms [57]. *Staphylococcus aureus* is the most frequent inciting pathogen and typically results in more severe illness. Milder clinical symptoms may be seen in infections caused by *Kingella kingae*; cases caused by this organism have been increasingly recognized in recent years, likely due to the development of more sophisticated molecular detection assays [17, 56].

**Fig. 7:**
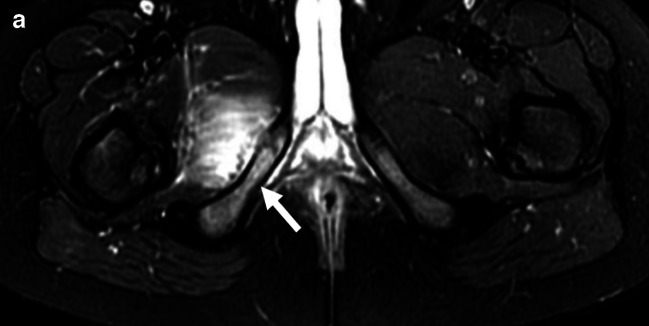
Osteomyelitis at the ischiopubic synchondrosis. A 4-year-old boy presented to the ED with right hip pain, limp, and fever. Initial radiographs (not shown) were unremarkable. **a **Axial T2W FS MRI demonstrates edema-like signal (*white arrow*) within the bone marrow and soft tissues adjacent to the right ischiopubic synchondrosis. **b** Axial T1W post-contrast image shows a small subperiosteal abscess (*dashed arrow*)

Hematogenous infection of the bone typically begins through bacterial seeding of a metaphysis or a metaphyseal equivalent location [17, 58]. Metaphyseal equivalents exist in the pelvis surrounding the tri-radiate cartilage, ischiopubic synchondrosis, and the sacroiliac joints (Fig. 7) [17]. Classically, it has been taught that these typical sites of infection can be accounted for by slow flow in looping terminal vessels in the metaphysis or metaphyseal equivalents, and that the relatively avascular portions of the physis in children older than 18 months tend to prevent the spread of infection across the growth plate to the epiphysis [59, 60]. Transphyseal vasculature is more robust in neonates, which allows for more frequent epiphyseal involvement [61]. However, more recent research indicates that transphyseal extension in the general pediatric population is more common than previously understood, possibly related to increasing virulence of certain bacteria (i.e., MRSA), due to widespread antibiotic use [62]. *K. kingae* osteomyelitis has a higher frequency of involvement of epiphyses and apophyses versus other organisms [63].

Imaging should focus on identifying the site of infection, evaluating for the presence of concurrent septic joint, assessing the extent of osseous involvement, and evaluating for the presence of collections requiring surgical drainage (which may include abscess, infectious tenosynovitis, and septic bursitis). The vast majority of infections occur in the lower extremities [64] and detection of concurrent contralateral disease typically does not alter clinical management [65]. Localization of infection is important as the diagnostic yield of aspirates and surgical cultures is greater than that of blood cultures [17]. Collections of sufficient size should be drained for local control [57].

Radiography is often the initial imaging modality in the acute setting [57]. While osseous changes of acute osteomyelitis are frequently radiographically occult, soft tissue changes (such as obliteration of fat planes) and joint effusions may be seen within the first few days; destructive osseous changes may take up to 10–12 days to become apparent (Fig. 6) [66]. Most importantly, radiography may exclude other causes of limp or extremity pain such as fracture or tumor [67].

**Fig. 8:**
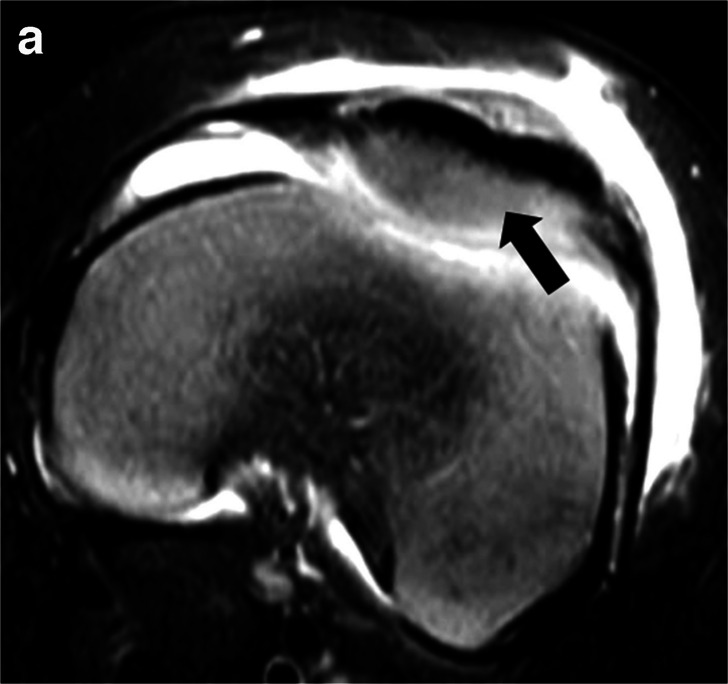
Unossified epiphyseal cartilage infection. A 14-month-old boy with septic arthritis and chondritis of unossified patellar cartilage. He presented to the ED with limp and fever. Axial T2W FS MR image (**a**) of the left knee shows subtle increased signal within the lateral aspect of the unossified patellar cartilage (*solid black arrow*). Axial T1W FS post-contrast image (**b**) shows lack of enhancement of this area (*solid black arrow*). The images additionally show a joint effusion with synovitis and inflammatory changes in the subcutaneous soft tissues

MRI has the highest sensitivity and specificity for diagnosing osteomyelitis [57]. It characterizes complications well, including subperiosteal collections, soft tissue abscesses, and sinus tracts, periarticular and intra-articular changes suggestive of septic arthritis, and vascular thrombosis. Osteomyelitis typically demonstrates T1 hypointense signal and hyperintense signal on T2-weighted sequences. However, abnormal signal on T1-weighted images may not be present in hematogenous osteomyelitis, which is more common in children [68]. Additionally, in young children with abundant red marrow, T1-weighted imaging becomes less reliable. Post-contrast enhancement is typically seen, though it may be heterogeneous or attenuated in the setting of concurrent ischemia [17]. Contrast is not necessary for the diagnosis of osteomyelitis on MRI in older children, though it is indicated in infants and young children due to the possibility of occult infection on non-contrast sequences in unossified epiphyseal cartilage (Fig. 8) [69, 70]. Contrast does improve the detection of abscesses and subperiosteal collections in the context of osteomyelitis, which may require surgical treatment [71]. Furthermore, contrast can be helpful in characterizing soft tissue infections and devitalized or ischemic tissue, thereby helping to guide debridement of necrotic tissue [69]. Challenges in administering contrast in children include the need for IV placement and sedation in young patients and ease of access in the acute setting [69, 72, 73], though this may be somewhat mitigated by the employment of new rapid MR protocols [74, 75] which are intended to be performed without contrast or sedation.

Another question that arises when performing MRI for musculoskeletal infection in children is what field of view to use. Larger fields of view, possibly including the whole limb, may be helpful in younger children without localized symptoms. For instance, the American College of Radiology appropriateness criteria for a limping child <5 years of age with concern for infection lists lower extremity MRI as the only usually appropriate imaging modality but alternatively lists MRI of the region as usually appropriate when symptoms are localized [76]. Different vendors and MRI imaging platforms will necessitate different coil usage but newer flexible blanket coils are often helpful for imaging an entire limb, while for more localized areas of concern the use of the smallest coil that covers the area of interest will help to improve image quality.

Scintigraphy, while able to diagnose osteomyelitis in cases where MRI is not possible, is limited in the emergency setting due to time constraints and availability, requires a relatively high radiation dose, and lacks the ability of MRI to detect concomitant areas of soft tissue involvement [17]. Like radiography, contrast-enhanced CT is insensitive to osseous changes of acute osteomyelitis though may characterize associated soft tissue or joint involvement. Non-contrast CT provides little utility and should be avoided. In this setting, ultrasound can diagnose soft tissue abscesses, subperiosteal collections, joint effusions, and vascular thrombosis but will not typically be able to diagnose osteomyelitis directly [57].

## Differential diagnosis for osteomyelitis

**Fig. 9:**
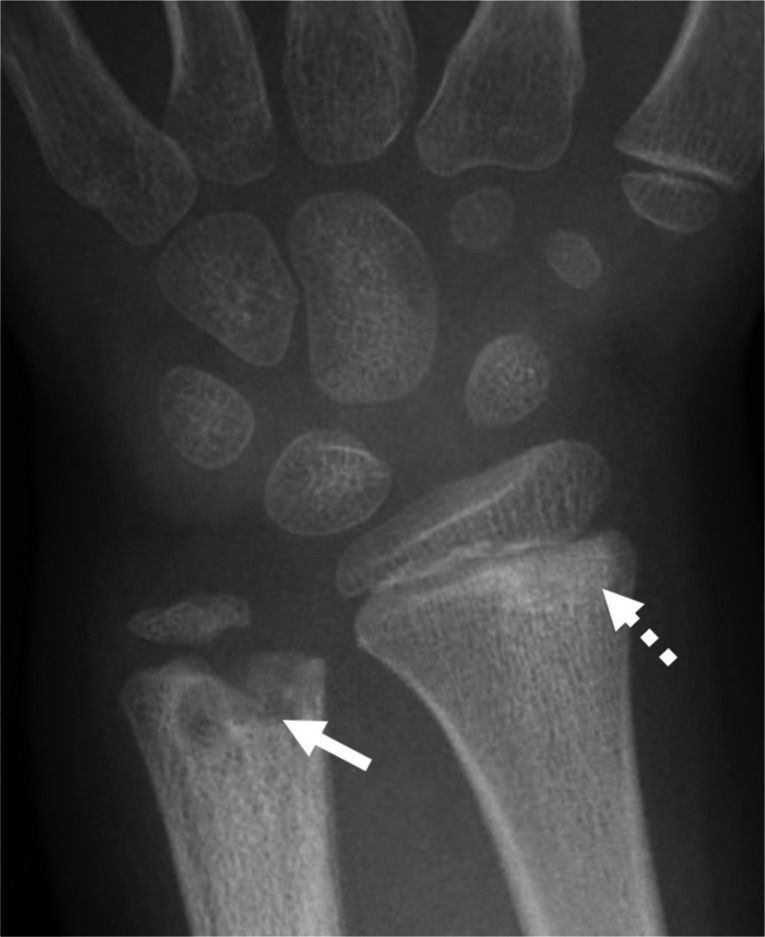
Chronic non-bacterial osteomyelitis (CNO). A 5-year-old girl presented with left wrist pain. The frontal radiograph of the left wrist shows a lytic lesion in the distal left ulnar metaphysis with adjacent sclerosis (*solid arrow*). A more subtle lesion is also present in the distal radial metaphysis (*dashed arrow*). The multifocality and metaphyseal location are suggestive of CNO which was later confirmed

**Fig. 10:**
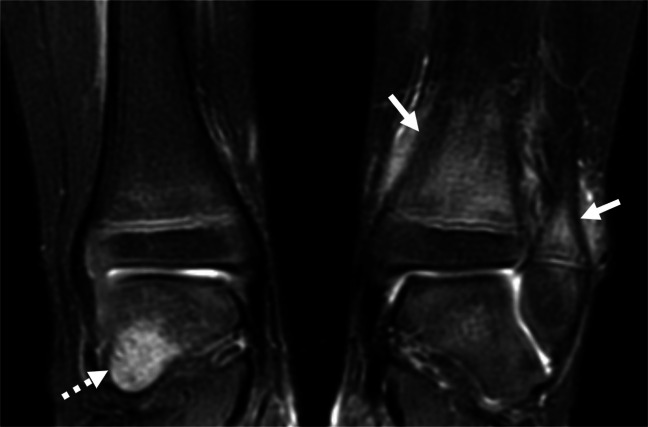
Chronic non-bacterial osteomyelitis (CNO) on MRI. A 4-year-old girl presented with pain and limp. Coronal short tau inversion recovery (STIR) MR image shows areas of edema-like signal within the distal left tibial and fibular metaphyses (*solid arrows*) with adjacent mild periosteal edema. Additionally, there is an area of edema-like signal within the right talus (*dashed arrow*). Other areas seen in the pelvis are not shown

When making a diagnosis of osteomyelitis, there are a number of key differential diagnoses one must also consider (Table 1) which include rheumatologic disease, malignancy, and metabolic disorders. Chronic non-bacterial osteomyelitis (CNO), also referred to as chronic recurrent multifocal osteomyelitis (CRMO), is an autoinflammatory disorder characterized by episodic and insidious pain in the affected bones [77, 78]. Active CNO lesions will present as lytic (typically metaphyseal) lesions, mimicking infectious osteomyelitis, neoplasm, and Langerhans cell histiocytosis (Fig. 9). MRI of the affected area is similarly non-specific, with T1 hypointense and T2 hyperintense signal which may precede radiographic evidence of disease (Fig. 10) [79]. Low-level surrounding soft tissue edema may be present in CNO, but abscess, sequestra, or fistulae should point to infectious osteomyelitis [79, 80]. In the reparative phase, sclerosis and hyperostosis predominate and edema will be less pronounced or absent [79]. Multifocal and symmetric lesions should raise the possibility of CNO (Figs. 10), as hematogenous osteomyelitis generally occurs in younger children and multifocality is less typical [80, 81]. The most commonly involved bones include the lower extremities (the tibia, femur, metatarsals), pelvis, clavicle, and spine [82]; the medial clavicle, mandible, and spine are particularly characteristic [81].

**Fig. 11:**
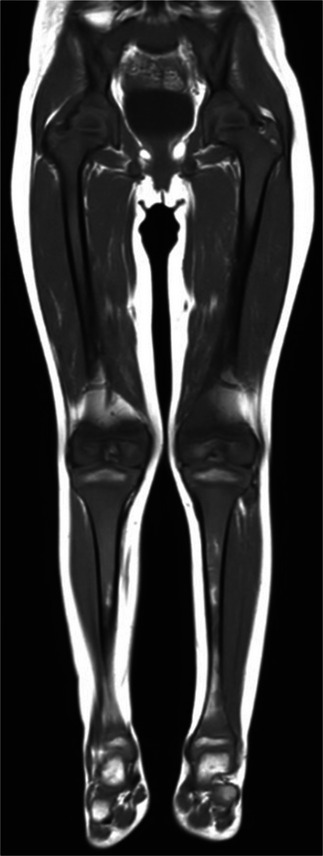
Leukemia. A 4-year-old girl with acute lymphocytic leukemia who presented to the emergency department with limp and fever. Coronal T1-weighted image of both lower extremities shows a diffuse marrow-replacing process with low signal intensity on the T1-weighted image throughout the bone marrow of the pelvis and the bones of both lower extremities. There was an increased marrow signal on a corresponding STIR image (not shown)

**Fig. 12:**
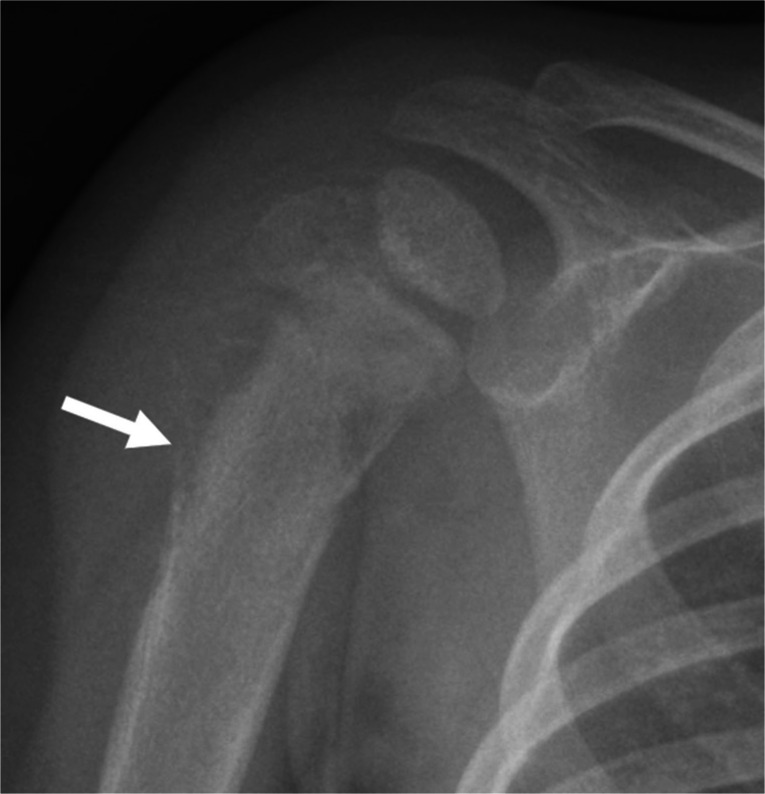
Metastatic neuroblastoma. A 1-year-old boy presented to the ED with 1 day of fussiness and decreased use of the right arm. Frontal radiograph of the right humerus shows a destructive lesion in the proximal right humerus with aggressive periosteal reaction (*arrow*). Follow-up MRI of the humerus (not shown) revealed an aggressive tumor and the patient was eventually diagnosed with metastatic neuroblastoma

Malignancy (particularly metastatic neuroblastoma and leukemia) should be considered whenever a focal, metaphyseal, or diffuse marrow-replacing process is encountered [83]. Leukemia is one of the most common childhood cancers and may cause lucent metaphyseal bands, pathologic fractures, and periosteal reaction on radiographs, which can appear similar to osteomyelitis [84]. However, on MRI, leukemia will typically manifest as diffuse hypointense signal on T1-weighted imaging and hyperintense signal on T2-weighted and STIR images, and demonstrate enhancement (Fig. 11), appearing much more confluent and diffuse than infection and without the associated soft tissue abnormalities [84, 85]. On MRI, metastatic neuroblastoma may demonstrate a similar diffuse infiltrative pattern although more nodular patterns of metastasis can occur. Metastatic disease (especially at the metaphyses) can also mimic the lytic and destructive changes of osteomyelitis on radiographs, but an enhancing soft tissue component evident on MRI should indicate the appropriate diagnosis (Fig. 12).

**Fig. 13:**
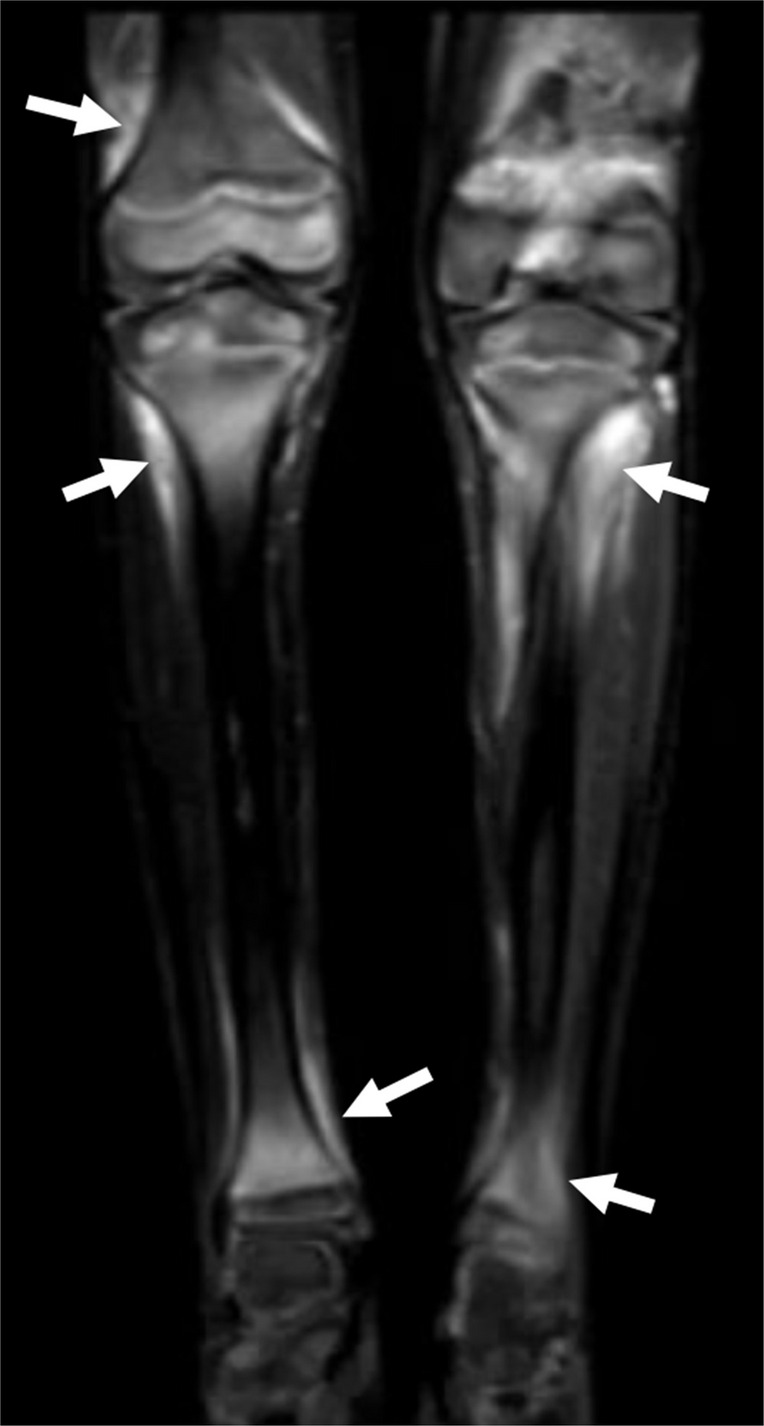
Scurvy. A 4-year-old boy with autism and a very limited diet presented with a limp. Coronal short tau inversion recovery (STIR) MRI of both legs shows bilateral edema-like signal within the distal femurs and proximal and distal tibias. There are adjacent areas of periosteal edema-like signal (*arrows*), which in the setting of scurvy may represent subperiosteal hemorrhage

Scurvy, a vitamin C deficiency, may be encountered in the emergency setting as a limp, refusal to use a limb, or refusal to walk. This diagnosis is increasingly seen in children with restrictive eating patterns (autism spectrum disorder, eating disorders) [86, 87]. Abnormal bone matrix and collagen production around sites of most rapid growth (such as the metaphyses) may mimic infectious osteomyelitis on MR with heterogeneous often hypointense T1 signal and associated T2 hyperintense signal (Fig. 13) [86, 87]. MR may also demonstrate periosteal edema thought to be related to subperiosteal hemorrhage [86].

## Conclusion

An enormous range of musculoskeletal pathology may be encountered in the emergency department. The spectrum of disease includes infectious, autoimmune, inflammatory, benign, and malignant conditions, though this review focused discussion on common and potentially morbid infectious conditions and their differential diagnoses. Infection can be seen anywhere in the body, though lower extremity involvement predominates in children. Radiography and ultrasound are first-line modalities in the initial workup of most conditions, helping to exclude common causes of pain and guide appropriate advanced imaging (typically MRI) if further imaging is required. MRI may also be needed in the setting of negative radiographs and/or ultrasound and ongoing high clinical suspicion.

## Data Availability

No datasets were generated or analysed during the current study
